# Population-Level Analysis to Determine Parameters That Drive Variation in the Plasma Metabolite Profiles

**DOI:** 10.3390/metabo8040078

**Published:** 2018-11-15

**Authors:** Mahmoud Al-Majdoub, Katharina Herzog, Bledar Daka, Martin Magnusson, Lennart Råstam, Ulf Lindblad, Peter Spégel

**Affiliations:** 1 Unit of Molecular Metabolism, Department of Clinical Sciences, Skåne University Hospital, Lund University, SE-205 02 Malmö, Sweden; mahmoud.al-majdoub@med.lu.se; 2 Centre for Analysis and Synthesis, Department of Chemistry, Lund University, 223 62 Lund, Sweden; katharina.herzog@chem.lu.se; 3 Department of Public Health and Community Medicine/Primary Health Care, The Sahlgrenska Academy, University of Gothenburg, 405 30 Göteborg, Sweden; bledar.daka@allmed.gu.se (B.D.); ulf.lindblad@gu.se (U.L.); 4 Department of Clinical Sciences, Lund University, 221 00 Malmö, Sweden; martin.magnusson@med.lu.se; 5 Department of Cardiology, Skåne University Hospital, Lund University, 205 02 Malmö, Sweden; 6 Department of Clinical Sciences in Malmö, Family and Community Medicine, Skåne University Hospital, Lund University, 205 02 Malmö, Sweden; lennart.rastam@med.lu.se

**Keywords:** metabolomics, glomerular filtration rate, insulin resistance, acylcarnitines, branched-chain amino acids

## Abstract

The plasma metabolome is associated with multiple phenotypes and diseases. However, a systematic study investigating clinical determinants that control the metabolome has not yet been conducted. In the present study, therefore, we aimed to identify the major determinants of the plasma metabolite profile. We used ultra-high performance liquid chromatography (UHPLC) coupled to quadrupole time of flight mass spectrometry (QTOF-MS) to determine 106 metabolites in plasma samples from 2503 subjects in a cross-sectional study. We investigated the correlation structure of the metabolite profiles and generated uncorrelated metabolite factors using principal component analysis (PCA) and varimax rotation. Finally, we investigated associations between these factors and 34 clinical covariates. Our results suggest that liver function, followed by kidney function and insulin resistance show the strongest associations with the plasma metabolite profile. The association of specific phenotypes with several components may suggest multiple independent metabolic mechanisms, which is further supported by the composition of the associated factors.

## 1. Introduction

Metabolomics, as other “omics” such as genomics, transcriptomics and proteomics, aims for a holistic view of biology in health and disease. In contrast to the transcriptome and proteome, which change gradually, the metabolome reacts instantly upon environmental changes, or in response to disease development [1]. Mass spectrometry (MS), coupled to various chromatographic techniques are commonly used in metabolomics methods [2]. Separations based on liquid chromatography (LC) combined with soft atmospheric pressure ionization have been increasingly applied in the last decade. One reason for this is the wide polarity range covered by the variety of stationary phase chemistries available in LC, allowing for separation of metabolites ranging from polar sugars to hydrophobic lipids. Currently, reversed phase chromatography is widely applied because of its high reproducibility and MS compatibility [2]. In a single analysis, metabolomics is, therefore, capable of detecting a broad range of physiological and pathophysiological processes. Consequently, metabolite profiling studies have linked circulating metabolite profiles to multiple metabolic pathologies, including cardiovascular [3,4], liver [5], and kidney disease [6], type 2 diabetes (T2D), and insulin resistance [7,8,9]. Recent studies indicated that the metabolite profile is more sensitive to disease development when compared to traditional clinical markers, which may allow for earlier detection of disease [10,11]. Most metabolomics studies focus on a single trait, and may produce an incomplete description of health status. Identified metabolite biomarkers are affected by the highly variable nature of the metabolome, and are not only impacted by multiple diseases, but also lifestyle-related factors, and anthropometric associations [1].

The aim of the present study was to identify the major determinants of the plasma metabolite profile. Using reversed phase ultra-high performance LC (RP-UHPLC) coupled to quadrupole time-of-flight MS (QTOF-MS), we measured plasma metabolite profiles of 2503 extensively phenotyped individuals. Our method covered a wide range of metabolite classes, including (phospho)lipids, amino acids, acylcarnitines, and bile acids. The metabolite profiles were converted into orthogonal metabolite factors and associated with 34 clinical covariates.

## 2. Results

### 2.1. Correlation of Metabolites

**Figure 1:**
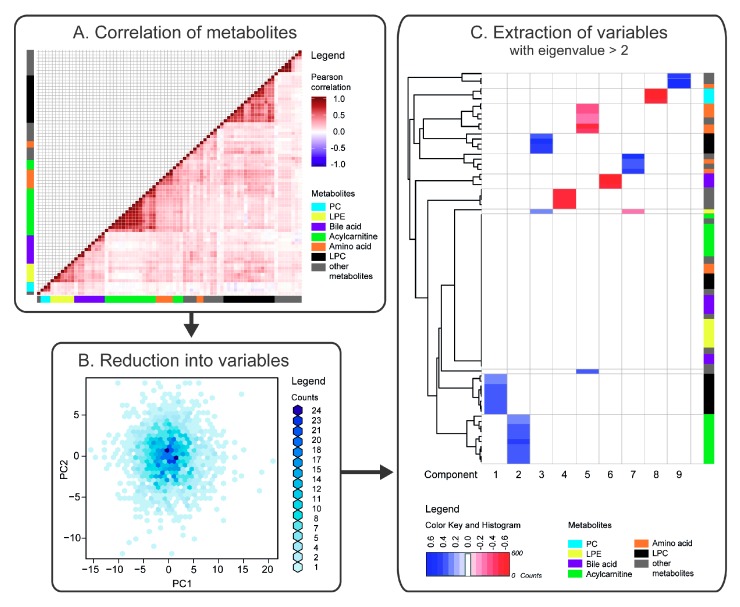
Schematic overview of correlation and principal component analyses (PCA) revealing independent clusters of metabolites. (**A**) Metabolite profiles are highly correlated. Pearson correlation coefficients for metabolite pairs (rows and columns) are shown. Several distinct clusters that correspond to biochemical pathways were observed, including phosphatidylcholine (PC, light blue), lyso-PC (LPC, black), lysophosphatidylethanolamine (LPE, yellow), acylcarnitine (green), bile acid (purple), and amino acid (orange) clusters. (**B**) PCA was employed to reduce the number of correlated metabolites by transforming them into uncorrelated metabolite factors. Density coloured scatter plot indicating the scores for the first two principal components (PC1 and PC2). The score plot hence indicates similarities and differences between the metabolite profiles of the subjects. The relation to the original variables, i.e., the metabolites, is described by the loadings (not shown). (**C**) The loading matrix contains non-zero values for all metabolites in all components. Hence, varimax rotation was conducted on the 18 principal components with eigenvalues > 1 to improve the interpretation of the factors. Heat map displays varimax-rotated loadings for the first 9 factors with eigenvalues ≥ 2, in which |loadings| ≤ 0.2, indicating only small contribution to the component, were coloured in white. Hence, the first component is largely composed of LPCs (black) and the second by acylcarnitines (green). Detailed graphs of Figure 1A,C are available in Figure S3.

We determined relative levels of 106 identified metabolites in plasma samples from 2503 individuals. These metabolites included amino acids, acylcarnitines, bile acids and (phospho)lipids. After removal of duplicates and metabolites that were detected in <85% of the individuals, 78 metabolites remained for further analysis. We first examined the composition of the metabolite profiles. Correlation analysis revealed several clusters of metabolites, which corresponded to distinct metabolite classes or pathways (Figure 1A). In particular, distinct clusters were observed for phosphatidylcholines (PCs), lyso-PCs (LPCs), lyso-phosphatidylethanolamines (LPEs), purines, amino acids, medium- and long-chain acylcarnitines.

### 2.2. Reduction of Metabolites into Uncorrelated Variables

To transform the correlated metabolites into uncorrelated factors, we analysed data using PCA. Thereby, the 78 correlated metabolites were reduced into 18 uncorrelated factors, which together explained 74.5% of the variation in the data (Figure 1B). Next, we used varimax rotation to enhance interpretation of the factors. The first 9 factors, with eigenvalues ≥ 2, are shown in Figure 1C. As metabolites that clustered along a particular component were generally related; specific metabolite classes could largely explain these components. The largest systematic inter-individual variation in the metabolite profiles was observed for LPCs (component 1, explaining 20.2% of the variation in the data), followed by medium-chain acylcarnitines (component 2, 10.6%), and long unsaturated LPCs (component 3, 6.8%). In addition, purines, caffeine, paraxanthine, and theophylline (component 4, 5.0%), branched-chain and aromatic amino acids (component 5, 4.3%), bile acids (component 6, 3.4%), LPE, phosphocholine, and amino acids (component 7, 3.2%), PC species (component 8, 3.0%), and uremic aromatic homo-monocyclic compounds (component 9, 2.5%) clustered along a particular component (Table S1 in Supplementary Materials).

### 2.3. Association of Metabolite Factors with Phenotypic Parameters

After reduction of the metabolite data into independent variables with metabolite class signatures, we investigated the association between the factors and clinical parameters. For this, we built linear models on the scaled data, and calculated standardized regression coefficients (*β*).

Prior to analysis, the 44 clinical covariates were screened for collinearity (Pearson |r| > 0.8) [12]. We used a relatively high threshold to keep the majority of clinically significant covariates. To focus on the most influential phenotypic traits, collinear parameters showing the weakest association with the first 10 principal components determined by principal component analysis (PCA) of the metabolite profiling data were excluded. The following clinical covariates showed collinearity: (1) body mass index (BMI), waist circumference, and weight; (2) cholesterol, low-density lipoprotein (LDL), and apolipoprotein B (ApoB); (3) free estradiol and estradiol; (4) testosterone, free androgen index (FAI), sex, and bio-available testosterone; (5) homeostasis model assessment of insulin resistance (HOMA-IR) and fasting insulin; (6) chronic kidney disease (CKD) and estimated glomerular filtration rate (eGFR). Of these variables, BMI, cholesterol, HOMA-IR, free estradiol, FAI and eGFR were used for association of phenotypic parameters to the metabolite factors.

**Figure 2:**
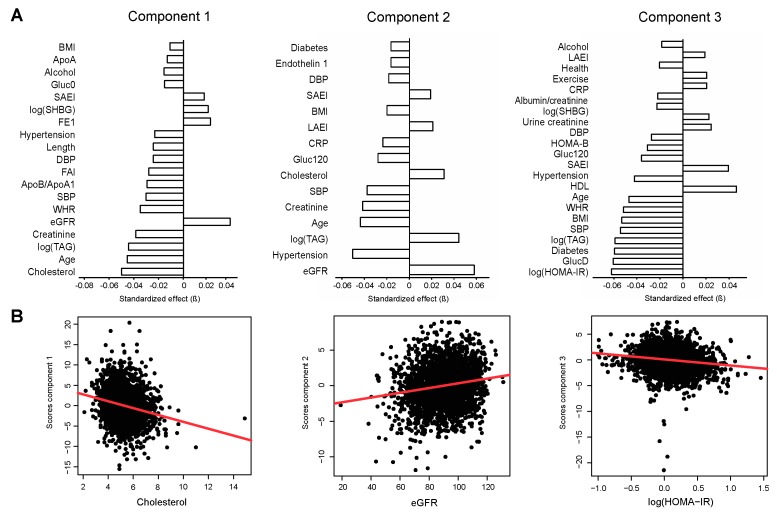
Principal components associate with distinct phenotypic features. (**A**) Associations of phenotypic traits with sample scores along the first three principal components (standardized regression coefficients, *β*; *q* < 0.05). (**B**) Cholesterol associates strongly with component 1 (left panel), eGFR with component 2 (middle panel), and HOMA-IR with component 3 (right panel). The linear regression line is shown in red. Abbreviations: Alcohol, alcohol intake gram/week; ApoA, apolipoprotein A1; ApoB, apolipoprotein B; CRP, c-reactive protein; DBP, diastolic blood pressure; eGFR, estimated glomerular filtration rate; Gluc0, fasting glucose; Gluc120, 120 min oral glucose tolerance test (OGTT) glucose level; HOMA-B, homeostasis model assessment of beta-cell function; HOMA-IR, homeostasis model assessment of insulin resistance; LAEI, large artery elasticity index; SAEI, small artery elasticity index; SBP, systolic blood pressure; SHBG, sex hormone binding globulin; TAG, triacylglycerides; WHR, waist/hip-ratio.

Component 1 associated strongly with measures of lipid metabolism, including cholesterol (*β* = −0.056, *q* = 1.20e^−27^), triacylglycerides (TAG; *β* = −0.050, *q* = 4.14e^−22^), and the apolipoprotein B/apolipoprotein A1 (ApoB/ApoA1)-ratio (*β* = 0.033, *q* = 2.73e^−10^) (Figure 2, left panel). These associations remained significant after adjustment for age and sex (cholesterol: *β* = −0.041, *q* = 5.91e^−15^; TAG: *β* = −0.034, *q* = 1.71e^−10^; ApoB/ApoA1-ratio (*β* = 0.017, *q* = 3.65e^−3^), and additional adjustment for BMI (cholesterol: *β* = −0.040, *q* = 7.73e^−15^; TAG: *β* = −0.033, *q* = 3.91e^−11^; ApoB/ApoA1-ratio: *β* = 0.017, *q* = 4.52e^−3^) (Table S2).

Component 2 associated with measures of kidney function, including eGFR (*β* = 0.059, *q* = 1.60e^−15^) and plasma creatinine (*β* = −0.042, *q* = 9.77e^−9^). In addition, blood pressure-related traits were associated with this component, including hypertension (*β* = −0.051, *q* = 3.14e^−12^), and both systolic (*β* = −0.038, *q* = 2.09e^−7^) and diastolic (*β* = −0.018, *q* = 2.10e^−2^) blood pressure (Figure 2, middle panel). After adjustment for age and sex, eGFR (*β* = 0.036, *q* = 5.62e^−9^), plasma creatinine (*β* = −0.037, *q* = 3.28e^−9^), and hypertension (*β* = −0.031, *q* = 3.19e^−6^) remained significantly associated with component 2, as well as after additional adjustment for BMI (eGFR, *β* = 0.037, *q* = 3.99e^−9^; plasma creatinine, *β* = −0.037, *q* = 2.57e^−9^; hypertension, *β* = −0.029, *q* = 1.26e^−5^) (Table S2). Notably, the association of eGFR and creatinine with component 2 were independent of hypertension (eGFR, *β* = 0.056, *q* = 5.14e^−9^; creatinine, *β* = 0.051, *q* = 6.42e^−8^).

Component 3 associated with measures of glycaemia, including HOMA-IR (*β* = −0.065, *q* = 3.92e^−2^), fasting glucose (*β* = −0.063, *q* = 5.53e^−12^), T2D (*β* = −0.062, *q* = 1.08e^−11^), 120 min oral glucose tolerance test (OGTT) glucose level (*β* = −0.037, *q* = 6.84e^−5^), and homeostasis model assessment of beta-cell function (HOMA-B, *β* = −0.032, *q* = 5.80e^−4^), as well as measures of obesity (BMI, *β* = −0.055, *q* = 7.58e^−10^; waist/hip-ratio (WHR), *β* = −0.054, *q* = 3.17e^−9^) (Figure 2, right panel). The associations remained significant after adjustment for age and sex (HOMA-IR, *β* = −0.058, *q* = 9.32e^−10^; fasting glucose, *β* = −0.049, *q* = 3.67e^−8^; T2D, *β* = −0.050, *q* = 3.67e^−8^; HOMA-B, *β* = −0.035, *q* = 3.43e^−4^; BMI, *β* = −0.0, *q* = 1.42e^−7^; WHR, *β* = −0.034, *q* = 1.45e^−6^). Furthermore, HOMA-IR (*β* = −0.032, *q* = 1.09e^−4^), fasting glucose (*β* = −0.038, *q* = 5.72e^−5^), T2D (*β* = −0.043, *q* = 1.12e^−5^), HOMA-B (*β* = −0.022, *q* = 3.66e^−2^), and WHR (*β* = −0.016, *q* = 2.96e^−2^) remained associated with component 3 after additional adjustment for BMI (Table S2).

Component 4 associated with eGFR (*β* = −0.086, *q* = 5.21e^−16^), FAI (*β* = −0.058, *q* = 2.16e^−7^), and age (*β* = 0.048, *q* = 1.33e^−5^), of which eGFR remained significantly associated with the component after adjustment for age and sex (*β* = −0.054, *q* = 9.89e^−9^), and additional adjustment for BMI (*β* = −0.054, *q* = 5.94e^−9^).

Both component 5 and 6 associated with sex- and age-related parameters. Component 5 associated with sex hormone binding globulin (SHBG; *β* = 0.168, *q* = 2.96e^−52^), FAI (*β* = −0.145, *q* = 3.2e^−38^), free estradiol (FE1, *β* = −0.049, *q* = 1.08e^−5^), and age (*β* = −0.088, *q* = 1.51e^−15^), and component 6 with age (*β* = −0.089, *q* = 1.04e^−11^), FAI (*β* = 0.090, *q* = 1.11e^−11^), and SHBG (*β* = 0.046, *q* = 7.64e^−4^). After adjustment for sex and age, however, component 5 associated strongly with HOMA-IR (*β* = −0.128, *q* = 6.69e^−29^), BMI (*β* = −0.123, *q* = 3.23e^−27^), SHBG (*β* = 0.104, *q* = 1.46e^−24^), high-density lipoprotein (HDL, *β* = 0.105, *q* = 2.92e^−21^), apolipoprotein A1 (ApoA1, *β* = 0.103, *q* = 5.07e^−21^), and WHR (*β* = −0.071, *q* = 1.06e^−15^), and these associations remained significant after additional adjustment for BMI (HOMA-IR, *β* = −0.063, *q* = 4.04e^−10^; SHBG, *β* = 0.061, *q* = 4.04e^−10^; HDL, *β* = 0.071, *q* = 2.44e^−10^; ApoA1, *β* = 0.085, *q* = 2.13e^−13^; WHR, *β* = −0.023, *q* = 7.79e^−3^).

Component 7 associated strongest with TAG (*β* = 0.131, *q* = 2.32e^−23^), and this association remained significant after adjustment for sex and age (*β* = 0.133, *q* = 1.23e^−26^), and additional adjustment for BMI (*β* = 0.122, *q* = 1.43e^−25^).

Component 8 was only associated with eGFR (*β* = 0.043, *q* = 3.72e^−2^), which did not remain significant after adjustment for sex and age.

Component 9 was associated with measures of obesity (WHR, *β* = 0.085, *q* = 7.99e^−8^; BMI, *β* = 0.072, *q* = 4.67e^−6^), alcohol consumption (*β* = 0.074, *q* = 4.67e^−6^), and eGFR (*β* = 0.061, *q* = 1.68e^−4^). These associations remained after adjustment for sex and age (BMI, *β* = 0.075, *q* = 3.70e^−6^; WHR, *β* = 0.052, q = 2.85e^−5^; alcohol consumption, *β* = 0.062, *q* = 8.86e^−5^; eGFR, *β* = 0.047, *q* = 4.72e^−4^). Both eGFR (*β* = 0.046, *q* = 1.53e^−3^) and alcohol consumption (*β* = 0.063, *q* = 1.86e^−4^) remained associated with this component after additional adjustment for BMI. Notably, the association of eGFR with component 9 was independent of alcohol intake (*β* = −0.045, *q* = 4.27e^−3^).

## 3. Discussion

In the present study, we established the main clinical covariates associated with differences in the plasma metabolite profiles in a cross-sectional sample of 2503 adult individuals. As multiple metabolites showed collinearity, we used PCA to reduce the large number of metabolites into independent components. The new orthogonal factors reflected different metabolite classes and pathways. Various LPCs contributed to component 1, which described the largest proportion of the variation in the data, and strongly associated with cholesterol and TAG levels, and the ApoB/ApoA1-ratio. Cholesterol levels are largely controlled by the liver, which is the major source of cholesterol and LDL via very low-density lipoprotein (VLDL) production, and also the major site of LDL catabolism [13]. In addition, TAG levels are largely controlled by the liver via synthesis and secretion of VLDL [14], and plasma levels of LPCs distinguish metabolically benign from malignant non-alcholic fatty liver [15]. Hence, our results suggest that liver function show the strongest association with the major differences in the plasma metabolite profiles measured in this study.

Medium-chain acylcarnitines mainly contributed to component 2. This component is associated with hypertension and kidney function, as approximated by eGFR. Hypertension is a proven risk factor for CKD [16], and the metabolome may therefore primarily reflect variations in eGFR that are secondary to hypertension. Our results confirm previous studies, which indicated higher acylcarnitine levels in CKD [17] and an inverse association with eGFR [18], which was suggested to be caused by impaired excretory function in the failing kidney [19]. In addition, renal excretion has been reported to be the primary route for acylcarnitine elimination [20]. Hence, the results in our study suggest that kidney function is strongly associated with differences in the plasma metabolite profiles.

In addition to component 2, eGFR also associated strongly with components 4 and 9. Component 4 was mainly contributed by purines, suggesting an inverse association between kidney function and circulating levels of purines. Increased intake of purines has been linked to hyperuricemia and gout [21], which in turn was associated with increased renal disease progression in animals [22]. In addition to its association with eGFR, component 9 associated with measures of obesity and alcohol consumption. Although BMI has been reported to be an independent risk factor for kidney disease [23], studies have shown that equations used to estimate GFR generally overestimate the parameter in obese individuals [24]. Whereas moderate alcohol consumption has been associated with an improved eGFR [25], high alcohol consumption has shown the opposite effect [26]. In our sample, only 2.2% of the individuals reported high alcohol consumption (≥4 servings of alcohol/day), whereas 77% reported none to moderate alcohol consumption. In addition, adjustment for BMI showed that the associations with eGFR were independent of obesity state. In line with this, a possible link between alcohol consumption and obesity has been disproved in large cross-sectional studies [27]. Furthermore, component 9 was enriched in uremic compounds, such as α-*N*-phenylacetyl-l-glutamine, hippuric acid, and p-cresol. While the former have been reported to be elevated in dialysis patients and can be reduced by increased dialysis frequency [28], elevated levels of p-cresol, a uremic toxin that is produced by bacteria in the intestine and secreted in the urine, has been linked to higher mortality in dialysis patients [29]. Although the association of eGFR with component 9 was independent of alcohol intake, a lifestyle-related effect cannot be excluded. Together, the results suggest that eGFR associates with two independent metabolic signatures, which are additionally associated with hypertension and a lifestyle-related variable, respectively.

Insulin is often considered the main determinant of the plasma metabolite profile [30]. However, the results in our study suggest that both liver and kidney function have a larger impact on the metabolite profile. Together with other measures of glycaemia and obesity, insulin resistance strongly associated with component 3. This finding is supported by similar results in studies that focused on factors associated with insulin resistance, revealing strong associations with higher components [31]. In our study, long unsaturated LPCs contributed significantly to component 3. Phospholipids containing unsaturated, long fatty acyl chains have been shown to be associated with a reduced risk for future T2D, whereas phospholipid species with shorter, saturated acyl chains were associated with an increased risk [32]. Overall, LPCs have been suggested to play a role in inflammation and endothelial dysfunction, conditions that are strongly linked to diabetes [33]. In general, several of the metabolites that comprised the main components presented in our study have been reported to be detrimental for human health, including medium- and long-chain acylcarnitines that have been reported to promote inflammation [34].

In the unadjusted model, component 5 associated with sex, SHBG and FAI. However, after adjustment for age and sex, it was strongly associated with HOMA-IR. Notably, this component was composed of branched-chain and aromatic amino acids, which are known to be higher in males [35] and to associate with insulin resistance and future risk of T2D [8,31]. Furthermore, indole, another metabolite associated with Component 5, is derived from dietary tryptophan in the gut [36]. The gut microbiota was recently reported as an important regulator of circulating branched-chain amino acids [37].

In this study, we used RP-UHPLC/QTOF-MS, a technique that is widely applied in metabolomics and that allows the detection of metabolites from multiple pathways, which have previously been associated with the investigated phenotypes. However, other techniques, covering other parts of the metabolome, may yield different results. Hence, a limitation of the study is the bias that potentially has been introduced by the choice of technique. Consequently, the number of detected metabolites remain limited in comparison to comprehensive studies involving several orthogonal platforms. Nonetheless, we cover metabolites from multiple classes, including amino acids, lipids and acylcarnitines, all of which are involved in central metabolic processes. Another possible limitation may be the composition of the sample; as the examined sample was collected in a specific region of Sweden, the application of these results to other populations remains to be examined. Genotype, lifestyle factors, dietary habits such as caffeine consumption, and medication are likely to influence the metabolome. We were not able to account for a number of these factors, as the frequency of medication among the population was low, and as information for several of these factors were not available. Finally, we have not performed any functional studies to investigate which tissue is responsible for the metabolite profiles we determined. Studying the link between tissue function and metabolite profiles is an interesting subject for future studies.

## 4. Materials and Methods

### 4.1. Subjects

The rationale of and the methodology used in the Vara Skövde Cohort within the Skaraborg Project have been described in detail [38,39,40]. In brief, a sample of 2816 subjects ranging from 30–74 years of age was randomly selected from the population census register of Vara and Skövde, Sweden, between 2002 and 2005. The sample was stratified for sex and age and intentionally oversampled in the group of 30–50 years of age, to obtain a mainly healthy, but representative sample of the population. Of these, we analysed 2507 individuals. Four individuals were excluded as they were lacking a substantial proportion of clinical data. Characteristics of the sample are shown in Table 1.

### 4.2. Clinical and Anthropometric Assays

Lifestyle and personal history questionnaires and anthropometric data were collected at baseline. Blood samples were collected after overnight fasting and stored at −80 °C until analysis. In total, 114 clinical covariates were available. These parameters included blood pressure, measured twice in supine position, artery elasticity by pulse wave analysis, and glucose and insulin levels as determined during an oral glucose tolerance test (OGTT). Lipoprotein profiles, inflammatory markers, albumin and creatinine levels were determined at the Clinical Chemistry Laboratory, Lund University. Hypertension was defined in accordance with international expert guidelines and JNC 7 criteria [43] or as on-going treatment for high blood pressure. T2D was defined after previous diagnosis of the disease or from the OGTT data according to World Health Organization (WHO) criteria [44].

After filtering for duplicates and variables with small variation (e.g., self reported diseases with low frequencies), 44 clinical covariates remained. For duplicates, clinically determined parameters were favoured over patient-reported information.

### 4.3. Metabolite Profiling

Metabolites were extracted from 40 μL blood plasma, spiked with internal standards, followed by analysis using RP-UHPLC/QTOF-MS as previously described in detail [30], with minor changes. In brief, metabolites were separated on an Acquity UPLC CSH C18 column (1.7 µm, 2.1 × 100 mm; Waters Corporation, Milford, MA, USA) using a 1290 Infinity UPLC connected to a 6550 iFunnel Q-TOF (Agilent Technologies, Santa Clara, CA, USA). Metabolites were eluted using a gradient composed of A, water with 0.1% (*v*/*v*) formic acid, and B, acetonitrile/isopropanol (75/25, *v*/*v*) with 0.1% (*v*/*v*) formic acid. The gradient was set to the following: 0–2 min, 2-20% B; 2–3 min, 20–40% B; 3–5 min, 40–95% B; 5–6 min, 95% B; 6–7.5 min, 95–2%; 7.5–10 min, 2% B. Analyses were performed in both positive (ESI+) and negative (ESI−) electrospray ionization mode. Metabolites were identified by MS/MS using in-house libraries and the MassHunter METLIN Metabolite PCDL (Agilent Technologies, Santa Clara, CA, USA). Leucine and isoleucine (leu/ile) were reported as a single metabolite, as these amino acids were not resolved with our method. All metabolites were manually integrated and confirmed in MassHunter Profinder B.06.00 Build 6.0625.0 (Agilent Technologies, Santa Clara, CA, USA). Only metabolites that were identified by MS/MS were used for data analysis; duplicates and metabolites with 100% missing values within a single batch were excluded. Within-batch variation was corrected for using the scores along the first principal component from PCA calculated in Simca P+ 12.0 (Umetrics, Umeå, Sweden) on the un-centred and unit variance scaled internal standards [45].

### 4.4. Statistical Analysis and Data Visualisation

All statistical analyses were conducted in R version 3.3.3 (2017-03-06). Metabolites were log2-transformed to approximate normal distributions. Samples were analysed over one year in batches of 150 samples. Hence, batch-to-batch variation was expected in the data. We used common variance compensation (ComBat, SVA package) to remove variation between batches [46], and used a dummy model matrix to not overfit the data (Figure S1). Missing data (≤15%) were imputed using k nearest-neighbour averaging (impute.knn, impute package) [47,48]. Correlation structures between the metabolites were visualized in heat maps using the packages heatmap.2 and hclust. Metabolite data were mean centred, scaled to unit variance, and analysed by PCA (princomp package; Figure S2). Subsequently, factors with eigenvalue ≥ 1.0 were further analysed by varimax rotation (varimax, stats package). Associations between factor scores and clinical covariates were evaluated by building linear models on the scaled data (lmFit, eBayes, limma package), and calculating standardized regression coefficients (*β*). For all analyses, significance was defined as *q* < 0.05 using multiple testing adjustments according to the false discovery rate method (p.adjust, stats package).

### 4.5. Compliance with Ethical Standards

All procedures performed in studies involving human participants were in accordance with the ethical standards of the institutional and/or national research committee and with the 1964 Helsinki declaration and its later amendments or comparable ethical standards. The Skaraborg Project was approved by the local ethical review board at Gothenburg University (Ö199-01). Informed consent was obtained from all individual participants included in the study.

## 5. Conclusions

The results in this study suggest that liver function is strongly associated with the differences in plasma metabolite profiles as determined by RP-UHPLC/QTOF-MS, followed by kidney function and glycaemic control. These results also indicate multiple independent components that are associated with the same phenotypic trait, which may suggest independent mechanisms determining the function of related organs. Whereas previous metabolomic studies have highlighted a vast number of biomarkers for various diseases, this study shows that a large number of these appear in a concerted manner. Moreover, several biomarkers previously detected in severe clinical conditions, e.g., in dialysis patients, could also be confirmed in the general population.

## Figures and Tables

**Table 1:** Characteristics of the study population.

Variable	Mean/Median/ Frequency	SD/IQR ^a^
N (men/woman)	1246/1257	-
Age (years)	47.8	11.8
Waist/hip-ratio (WHR)	0.9	0.1
Length (cm)	172.2	9.5
Body mass index (BMI; kg m^−2^)	26.9	4.6
Fasting plasma glucose (Gluc0; mmol L^−1^)	5.4	1.1
120 min OGTT plasma glucose (Gluc120; mmol L^−1^)	5.6	2.2
Homeostasis model assessment of insulin resistance (HOMA-IR)	1.2	0.8–1.9
Homeostasis model assessment of beta-cell function (HOMA-B)	59.8	42.6–84.2
Type 2 diabetes (T2D) (yes/no; %)	141/2359; 5.6%	-
Small artery elasticity index (SAEI; mL mmHg^−1^)	7.4	3.5
Large artery elasticity index (LAEI; mL mmHg^−1^)	16.4	5
Pulse (min−1)	63.7	8.4
Systolic blood pressure (SBP; mm Hg)	121.8	16.9
Diastolic blood pressure (DBP; mm HG)	70.3	10.1
Hypertension (yes/no; %)	363/2140; 14.5%	-
Apolipoprotein A1 (ApoA1; g L^−1^)	1.7	0.3
ApoB/ApoA1	0.6	0.2
Triacylglycerides (TAG; g L^−1^)	1.1	0.8-1.6
High density lipoprotein (HDL; mmol L^−1^)	1.3	0.3
Cholesterol (mmol L^−1^)	5.3	1.1
Creatinine (mol L^−1^)	78.8	13.9
Albumin/creatinine-ratio	0.3	0.2–0.5
Urine creatinine (mol L^−1^)	12.2	5.9
Estimated glomerular filtration rate (eGFR; mL min^−1^ 1.73 m^−2^)	89.9	14.4
Testosterone (nmol L^−1^)	22.7	2.4–46.0
Estradiol (nmol L^−1^)	4.4	2.9–6.9
Sex hormone binding globulin (SHBG; nmol L^−1^)	37.5	26.7–52.2
Health (1/2/3/4/5) ^b^	434/1380/581/76/7	-
Exercise (1/2/3/4) ^c^	171/1432/748/73	-
Smoking (yes/no; %)	454/2040; 18.2%	-
Alcohol (g/week)	25.2	6.3-59.7
C-reactive protein (CRP; mg L^−1^)	1.3	0.7–2.7
Endothelin 1 (pg mL^−1^)	2.4	1.3
Cortisol (nmol L^−1^)	2	2.0–3.5

^a^ Quartile 1–quartile 3; ^b^ Health was defined as (1) excellent, (2) good, (3) fair, (4) poor, and (5) very poor [41]; ^c^ Level of exercise was defined as (1) inactive or mostly inactive, e.g., reading or watching television; (2) slightly active, at least 4 h of activity, e.g., spare time walking, cycling, gardening including walks or cycling to or from work; (3) moderate, less strenuous, e.g., exercise for at least 2 h a week, such as jogging, swimming and tennis; (4) strenuous, e.g., intensive jogging, swimming and tennis several times a week [42].

## Supplementary Materials

The following are available online at http://www.mdpi.com/2218-1989/8/4/78/s1, Figure S1: Additional information of data pre-treatment, Figure S2: PCA scree plot, Figure S3: Detailed graphs of Figure 1A,C, Table S1: Overview of the first 9 factors with eigenvalues ≥ 2 from PCA, Table S2: Overview of the results from the linear models to assess the association of metabolite factors with phenotypic parameters.
