# Supplementary material for: Population-Level Analysis to Determine Parameters That Drive Variation in the Plasma Metabolite Profiles
Source: Metabolites. 2018 Nov 15;8(4):78. doi: 10.3390/metabo8040078 (PMC6316279; doi:10.3390/metabo8040078)
Supplement: Supplementary file 1 [file metabolites-08-00078-s001.zip › Supplemental Material_Supplemental Fig. 1 to Fig. 3.pdf]

Supplemental Figures

Article title: Population-level analysis to determine parameters that drive variation in the plasma metabolite profiles

Journal: Metabolites

Author names: Mahmoud Al-Majdoub, Katharina Herzog, Bledar Daka, Martin Magnusson, Lennart Råstam, Ulf Lindblad, Peter Spégel

Affiliation and Email address of the Corresponding author: Peter Spégel, Department of Chemistry, Centre for Analysis and Synthesis, Naturvetarvägen 14, SE-223 62 Lund, Sweden. Email: peter.spegel@chem.lu.se

TABLE OF CONTENT

Supplemental Figure 1

Supplemental Figure 2

Supplemental Figure 3

Supplemental Figure 1

A

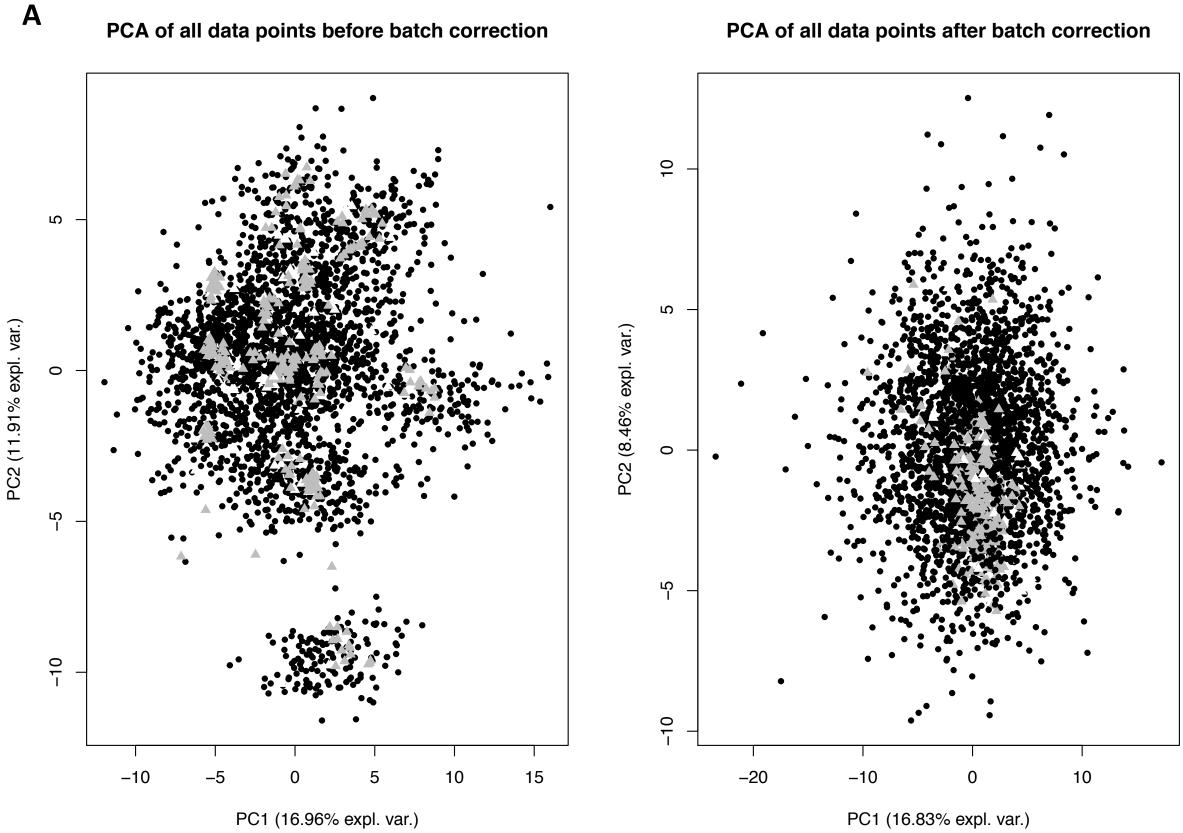

B

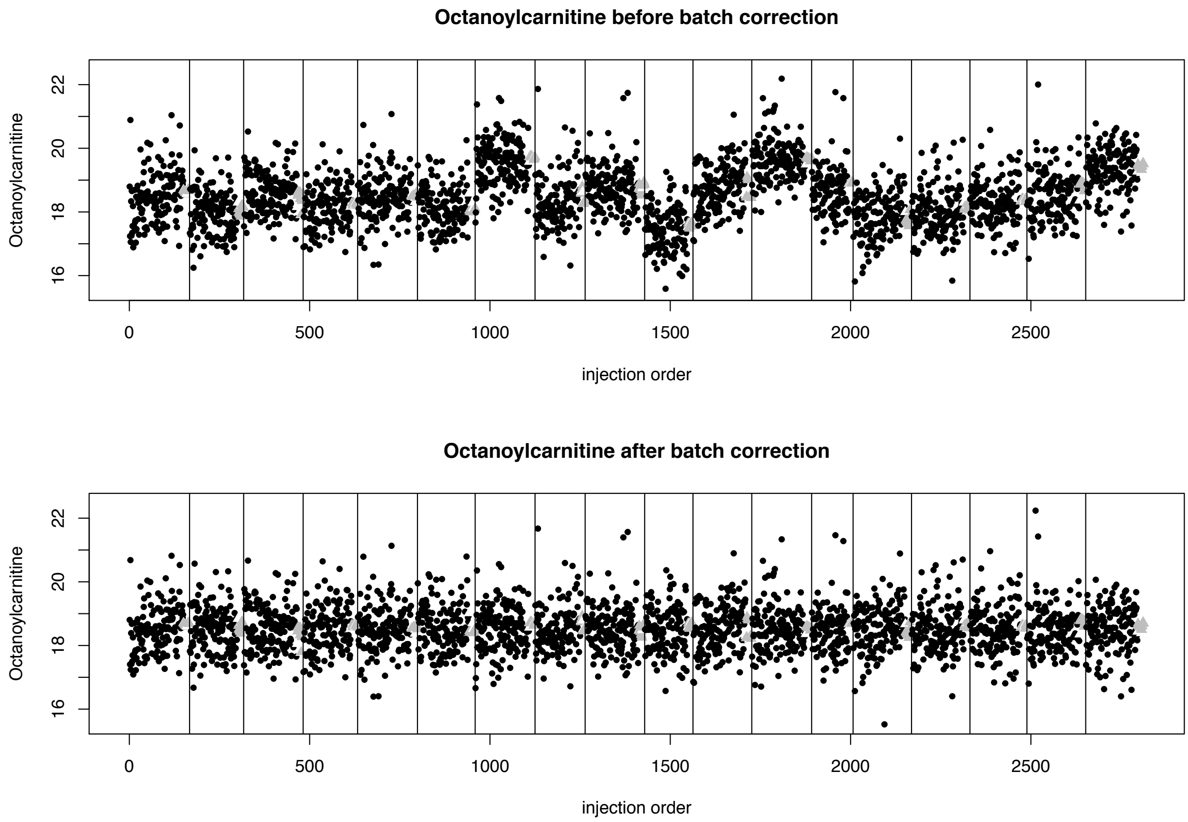

Supplemental Figure 1. (A) Score plot of the first two principal components (PC1 and PC2) from a principal component analysis calculated on the metabolite data before (left) and after application of batch correction using the ComBat method (right). (B) Scatter plot of peak areas for octanoylcarnitine before (upper panel) and after application of batch correction (lower panel). Samples are sorted by injection order, and vertical lines separate batches. Pooled samples of all plasma samples injected within each batch are indicated in grey triangles.

Supplemental Figure 2

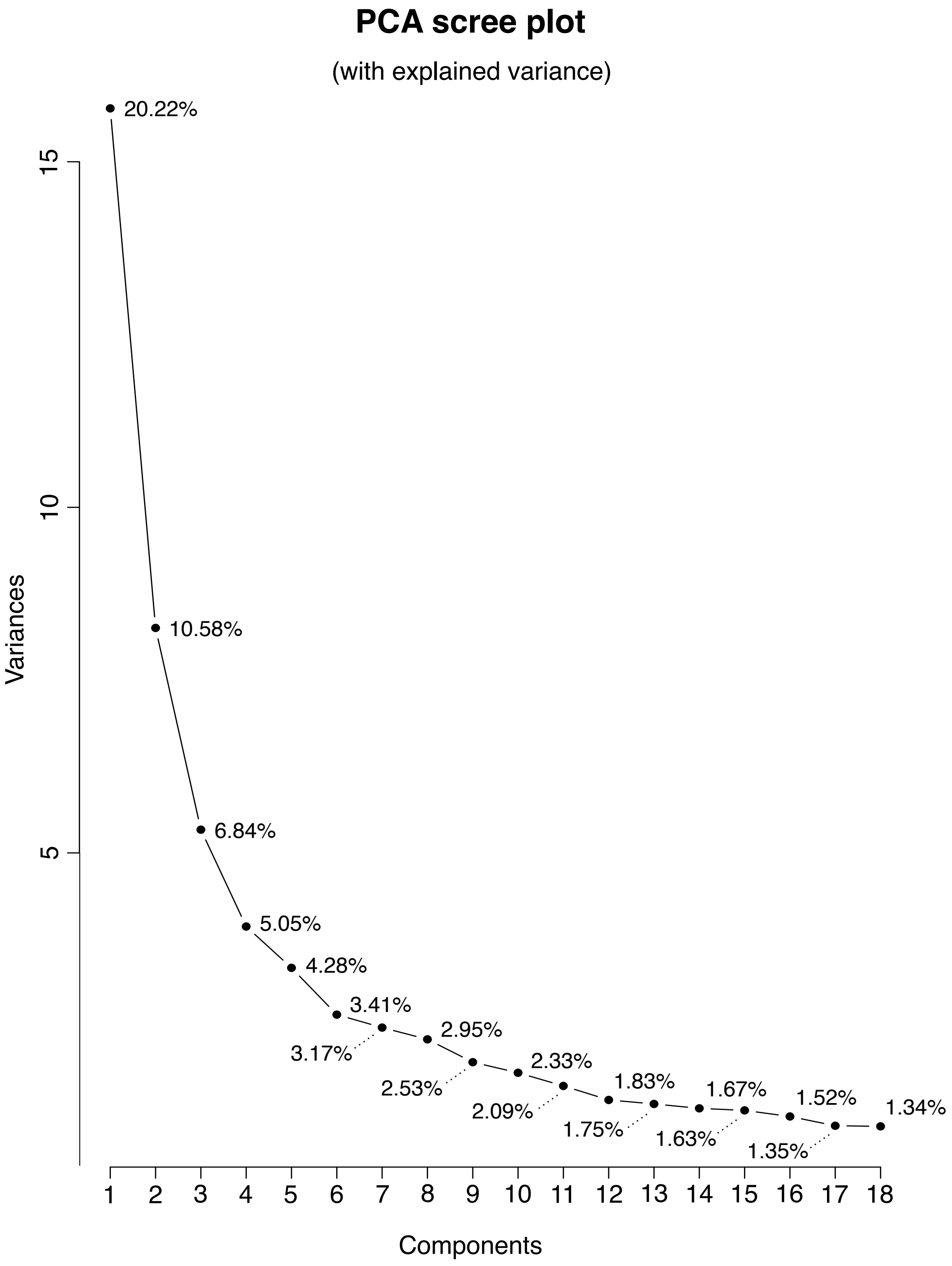

Supplemental Figure 2. PCA scree plot indicating the explained variance of the first 18 components.

Supplemental Figure 3

A

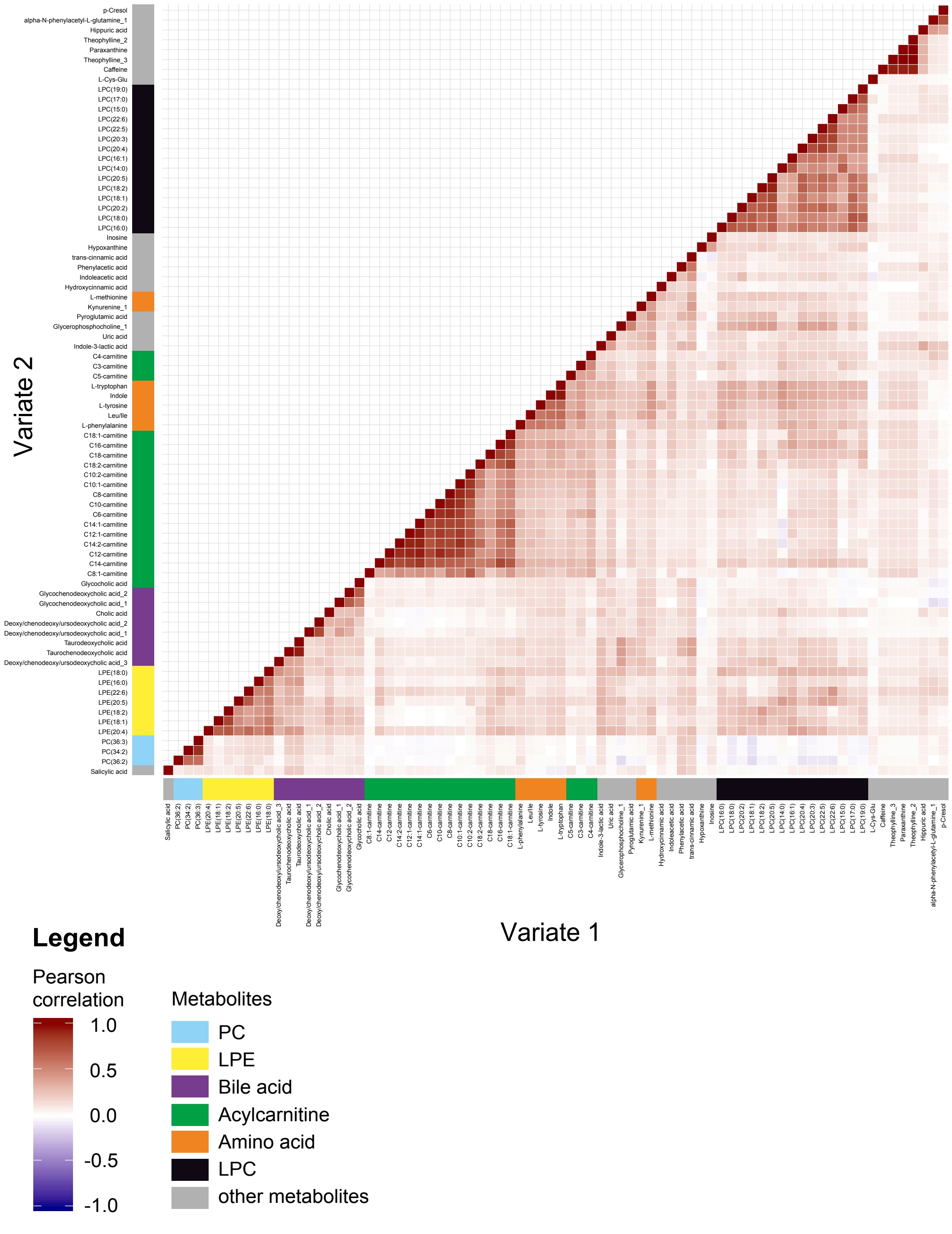

B

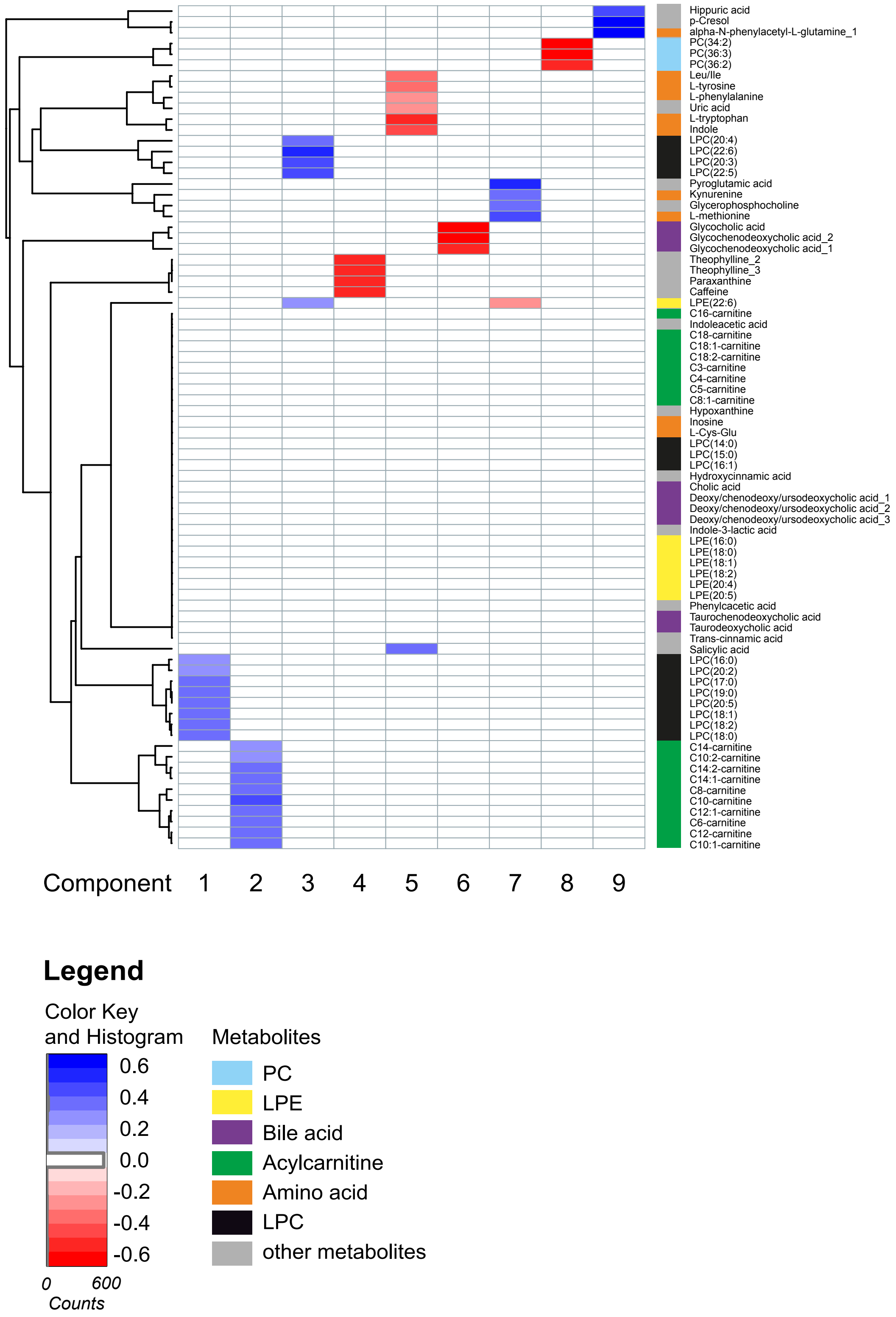

Supplemental Figure 3. Enlarged graphs of (A) Figure 1A and (B) Figure 1C.
